# Simon Fraser University Speech Error Database – English (SFUSED English): Methods and Design

**DOI:** 10.5334/joc.440

**Published:** 2025-04-01

**Authors:** John Alderete

**Affiliations:** 1 Linguistics, Cognitive Science, Simon Fraser University, Burnaby, V5A 1S6, CA

**Keywords:** Speech errors, language production, phonetics, phonology, morphology, syntax, semantics, rating data, data quality, data science, methodology

## Abstract

SFUSED English (Simon Fraser University Speech Error Database – English) is the first large scale database of speech errors developed from audio recordings of spontaneous speech. This article describes the structure of the database and the standards used to construct it, including collection and classification methods, record mark-up, data quality measures, and adherence to standard practices in psycholinguistics and English linguistics. Additional information on these methods and the entire database are available on the OSF repository: https://osf.io/8c9rg/.

## Introduction

The Simon Fraser University Speech Error Database – English (SFUSED English) is a multi-purpose database designed to support both language production and linguistic research. It contains over 10,000 speech errors collected from spontaneous speech in English, and 8,949 of these errors were collected from publicly available audio recordings. The creation of an English database began in 2014 out of a perceived need to address some of the problems in data collection in past research (Schütze & Ferreira 2007), and this led to a spin-off database, SFUSED Cantonese (Alderete & Chan 2018), the first speech error study of this under-studied language. The general methodology used for both databases is documented in some detail in Alderete and Davies (2019), which reviews and assesses the benefits of collecting speech errors from audio recordings and with multiple trained data collectors. The methods specific to SFUSED Cantonese, such as linguistic assumptions for Cantonese, have been described in Alderete (2024), but this work also gives a general account of project management and processing assumptions that apply to SFUSED English. The current work focuses on the methods specific to SFUSED English that have not yet been covered in these works. It documents the standards used to encode English linguistic structure, psycholinguistic measures such as rating data, and data quality measures specific to this database.

## Methods overview

As a **definition of speech errors**, we assume that an error is “an unintended, nonhabitual deviation from a speech plan” (Dell 1986). They are typically sound or word mis-selections that native speakers produce with some regularity, but also include morpheme mis-selections, syntactic errors in word order, word additions and deletions, and unintended realizations of morpho-syntactic features (Shattuck-Hufnagel 1979; Stemberger 1982; Stemberger 1982/1985). This definition excludes false starts, idiolectal or dialectical variants, casual speech phonology, and changes in the speech plan mid-stream. The methods folder in the OSF repository contains detailed documentation of English dialects, casual speech phonology, and the specific rules we used to exclude these habitual speech phenomena from the data collection.

As a **classification system**, speech errors are categorized using a standard system of grouping speech errors based on the linguistic unit (or level), the type of process, and the direction of source units (Dell 1986; Shattuck-Hufnagel 1979; Stemberger 1993). For example, phonological substitutions involve a mis-selection at the level of sounds, substitution as a process, and can be cross-classified as anticipations, perseverations, exchanges, etc. We also include a Mastertype field that bundles these major class fields into pre-compiled packages (e.g., lexical substitution, sentence blend, etc.) that simplify searches and also correspond roughly to the failures in language production processes commonly assumed in theoretical models (e.g., Bock and Levelt (1994)).

Speech errors in SFUSED English were collected by 14 trained data collectors. Their month-long **training** involved an introduction to the psycholinguistics of speech errors, practice with English phonetic transcription, and three extended listening tests in which trainees were instructed to collect speech errors from pre-screened recordings and given feedback on correctly and incorrectly submitted data. All of the materials used to train data collectors are in the training folder in the OSF repository.

Finally, general **data quality** can be assessed with the information in Table 1. A relatively high percentage of sound errors is considered a good measure of representativeness (Pérez et al. 2007). Sound errors were the most common in the database and much more common than word errors (39.18%), the next highest class. There is a relatively low percentage of exchange errors and a high percentage of phonotactic violations in sub-lexical errors, which also indicate high data quality (Alderete & Davies 2019). We can assess sample coverage by comparing the minutes per error (i.e., the time elapsed on average during which one error is observed) and compare that with the estimated frequency of errors in SFUSED English from Alderete and Davies (2019). This analysis shows that our data collectors missed many errors, with about a 1 in 3 rate of collection. Compared to an MPE of 0.84 for SFUSED Cantonese, these numbers indicate that the Cantonese database has better sample coverage. However, the MPE for SFUSED English is less than half of the MPEs of two other speech error studies based on audio recordings (see Alderete and Davies (2019) for details), which suggests some improvement over past research in reducing missed errors.

**Table 1:** Data quality measures.


MEASURE	SFUSED ENGLISH

Percentage of sound errors	50.78%

Percentage of exchange errors (all types)	1.2% (cf. 0.57% offline)

Percentage phonotactic violations	5.46%

Minutes per error (MPE)	2.43

Estimated frequency of errors	48.5 s (or less)


SFUSED English has been compared with English speech error collections of similar sizes, such as the Stemberger and MIT-Arizona collections (Alderete & Davies 2019). To summarize, the differences in methods (i.e., availability of audio, multiple trained data collectors, and a verification stage) have a large impact on data composition and quality, demonstrating the robustness of SFUSED English to perceptual biases compared to other collections. The primary use of audio means that SFUSED English differs from phonetic studies that collect articulatory measures of speech errors (Frisch 2007; Goldstein et al. 2007). However, the audio backup does support some types of speech analysis, including a recent investigation of voicing duration in phonetic blends (Alderete et al. 2021). Finally, SFUSED English was developed in a lab-based learning community at Simon Fraser University, and so it differs from a recent innovation that accelerates research through third party crowd-sourcing of data collection (Vitevitch et al. 2015).

## The database

The database can be approached from three different lenses: data tables, interfaces, and fields. First, SFUSED English is constituted by three **data tables** in which rows are entries and columns are attributes of these entries. The speech errors data table is the core of the database, but there are two other important tables, one for the audio recordings and one for the podcast series these recordings are from. The data in these tables can be related using a linker field to make a relational database, but in practice they are accessed as separate flat databases.

Our team initially collected 1,112 speech errors from direct in-person conversations (which were not recorded) and then switched to collecting errors exclusively offline from audio recordings because of their benefits for data quality and discovery (Alderete & Davies 2019).

The audio **data sources** were drawn from 472 different episodes of publicly available podcast series, such as *The Astronomy Cast* and *Rooster Teeth*. The ten series that we examined were pre-screened to ensure each series contained high production quality, gender balance in commentators, diversity in media genre, and a high percentage of unscripted spontaneous speech. While there were a few guest speakers with foreign accents, almost all the podcast commentators used a standard English dialect common in the US and Canada, which was typically a variety of the Midland American English dialect. The combined length of these recordings, after excluding set scripts and music, was approximately 360 hours. The dataRecordings data table in the OSF data folder has a row for each of these recordings and gives the following information about them: episode length, the speakers, and linguistic notes on dialectal and idiolectal speech features. The dataSeries data table in the same folder has a row for each podcast series and lists its URL, copy-right and licensing information, series format, and common topics. The README file in the OSF file directory gives an overview of all this information.

**Figure 1:**
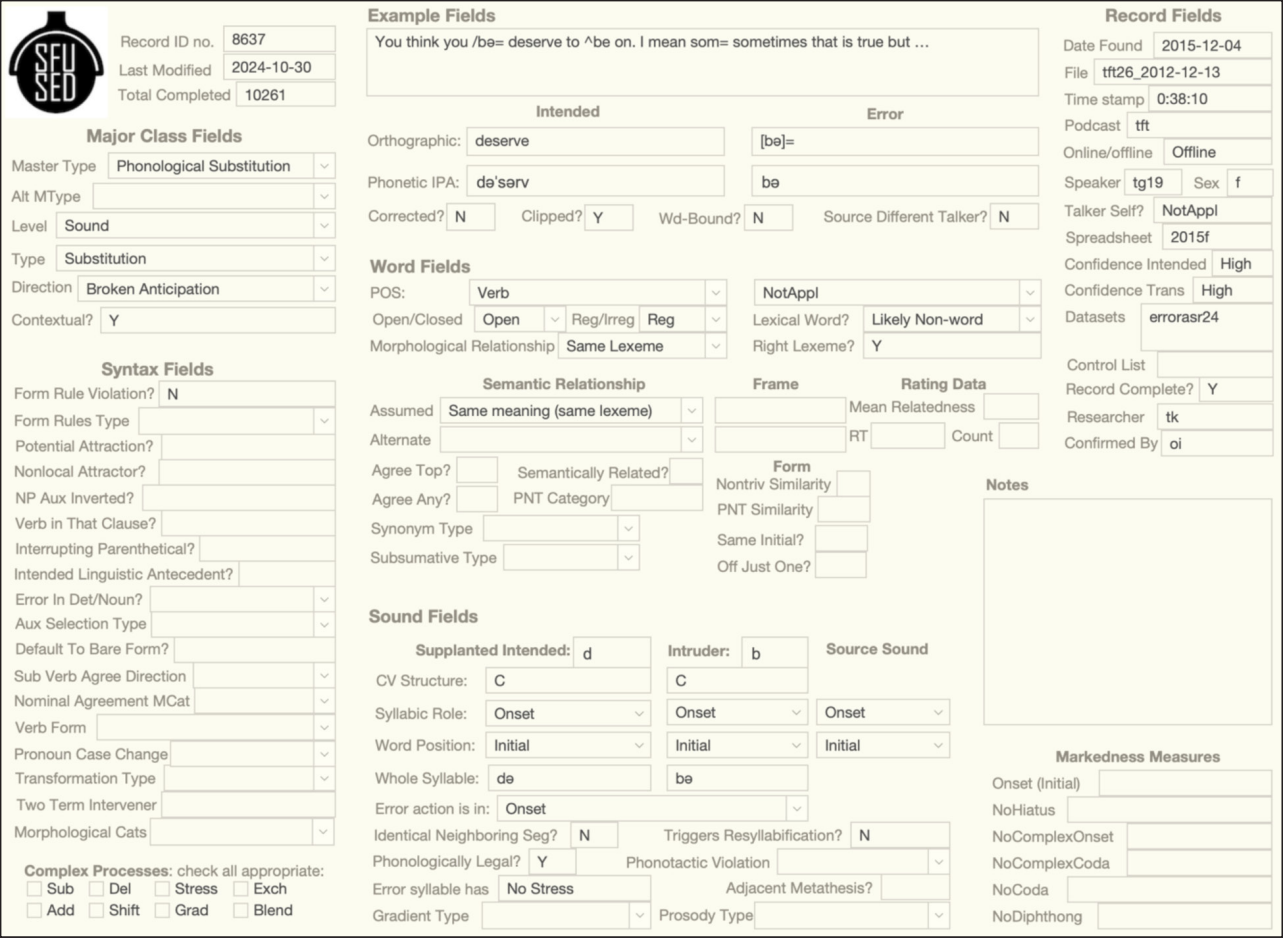
Longform interface of phonological substitution error.

The database can also be approached with two **interfaces**: a longform interface that displays all the information about a single speech error, and a dimensions interface that visualizes the distributions of speech errors in the entire database. The longform interface shown in Figure 1 is a layout created in FileMakerPro that displays the longform of an error at the top-center and arranges its field values by type. While the longform interface is good for data input and examination of individual examples, the dimensions interface is preferred for research projects because it supports rapid extraction of contingency tables and confusion matrices. The OSF repository includes a FileMakerPro database as a longform interface and a Tableau workbook as a dimensions interface. However, new interfaces can be created in a variety of platforms (e.g., Python pandas, R) using the raw data also available in the repository.

Finally, the database can be viewed as a large data table with rows for speech errors and columns for 113 **fields**. These fields are grouped by class, as shown by the sections in Figure 1. The Example fields in the top-center display the longform of the speech error, and these fields break it down into a set of observable traits, such as if the error is corrected or not. Notice that the Example fields break the error down into intended and error terms, and that these terms are at least as large as words. This distinction is continued below in the Word fields, which provide lexical information about both intended and error terms. Likewise, the Sound fields analyze the sub-lexical portions of the error and give attributes of the intended, error, and source sounds. The Major Class fields appear top-left and provide the principal classification of the error (see above). Syntax fields on the bottom left give attributes about morphological, morpho-syntactic, and syntactic speech errors, and Record fields on the right list facts about the record itself, like the speaker label, audio file, and time stamp. Full descriptions of all fields are given on the wiki of the OSF project.

To illustrate some of these fields, consider how they can be used to test well-known psycholinguistic findings. Alderete and Davies (2019) used two word fields, Intended part-of-speech and Error part-of-speech, to confirm the Category Constraint, that is, the fact that intended and error words tend to be the same part of speech (Bock 1982; Garrett 1975). Similar searches on phonological structures can be used to investigate sound-level principles. For example, the phonological similarity effect (Shattuck-Hufnagel & Klatt 1979) can be assessed by making a confusions matrix with Supplanted Intended and Intruder sounds, as illustrated in detail in Alderete (2024).

To read the longform of a speech error and process it programmatically, it is necessary to understand the basic **mark-up**, summarized in Table 2. The error is marked with a “/” prefix, and if the error unit is larger than a word, then the last word of the error phrase has a “#” suffix. A useful way to think of this mark-up is that if the intended word or phrase replaced the error in this position, a speech error would not have occurred. Table 2 also shows how source words (i.e., the words providing the material of the error) and trigger words are prefixed, pauses and interruptions, clipped words, and speaker turns.

**Table 2:** SFUSED Mark-up in longform entries.


/	Prefixed to error word or phrase

#	Suffixed to the end of a multi-word error term

^	Prefixed to source words

$	Prefixed to trigger word in deletions

…	Trailing speech at beginning or end of longform entry

=	Suffixed to clipped words (i.e., not completed)

A:, B:	Used for speaker turns with more than one speaker

xxx	An interruption in the speech stream

,	Pause without interrupting speech


The database is designed around the idea that most speech errors can be encoded as a combination of level and type; that is, a deletion, addition, or substitution of some linguistic unit. Some speech errors, however, are not amenable to such an analysis and therefore require **adaptations** and context sensitive use of certain fields. In order to represent the structure of a word blend, for example, the intended field is a word pair rather than a single word, and the error itself is the fusion of these two words. Likewise, word shifts require documentation of both location of the shifted word in the error and its intended location. These so-called two term errors thus have two /-prefixed error items to locate both terms, a trick we also use for exchange errors. These adaptions are explained in more detail for SFUSED Cantonese in Alderete (2024), and the ideas are the same for this database.

## Linguistic assumptions

Some Example and Sound fields use **phonetic transcription**. The general rule in the Example fields is to encode the intended and error terms orthographically, unless the production of these terms cannot be straightforwardly recovered from spelling, in which case they are transcribed using the International Phonetic Alphabet (IPA) for English (as in Ladefoged (2006)). Two Example fields and the Sound fields also use the IPA in all cases. Spontaneous speech sometimes exhibits less than ideal productions, and we use a set of conventions from Stoel-Gammon (2001) to transcribe fuzzy speech, ambiguous segments, segments that transition from one to another category, and weakly articulated sounds. The speech corpus also includes some non-native sounds, which are transcribed with standard IPA symbols. Stress is also transcribed using IPA convention, though to standardize, each word is given a word level primary and secondary stress. This practice is straightforward for single words and compounds, but longer phrases require additional phonetic analysis if phrasal stress is desired. Another standardization is that syllabic sonorants are represented as a VC structure, as in [bʌtər] for *butter*. The phonetic toolkit in the OSF methods folder explains all of these assumptions in more detail.

Sound fields analyze the **syllabic structure** of intended, error, and source sounds by assigning them a syllabic role. These roles follow conventional wisdom on English phonology, employing established formal accounts of English syllabification (Giegerich 1985; Jensen 1993; Selkirk 1982). The appendix of Alderete and Tupper (2018) explains in detail how segments are assigned to onset, nucleus, and coda positions, and the appendices that are possible word initially and finally. This system of syllabification in turn supports two sound fields: Phonologically Legal?, which classifies errors by whether they obey English syllabification, and Phonotactic Violation, which classifies phonotactic violating errors by type.

Sound errors that involve phonological deletion or addition affect **markedness**, and this factor is assessed using a set of markedness measures inspired by speech error studies of aphasic patients (Béland & Paradis 1997; Blumstein 1973). These errors are assessed by six surface-oriented markedness constraints that determine if the deletion or addition satisfies or violates specific markedness conditions. For example, the deletion error *glow* → *go* reduces the initial *gl* cluster and therefore reduces the markedness of this form by satisfying the constraint NoComplexOnset. These constraints overlap with surface phonological constraints used in Optimality Theory (Prince & Smolensky 1993/2004), and so they support questions about how speech errors inform phonological theory.

Stems can appear as part of **compounds or phrases**, and since their wordhood is important to fields that assess linguistic level, we require a method for distinguishing compounds and phrases. We follow standard methods (e.g., Haspelmath and Sims (2010)) in using a combination of facts as well as the analysis given in the Merriam-Webster Dictionary of English. Compounds like *yellow jacket* in general exhibit more idiomatic meaning, have compound stress, and fail on the replacement by *one* test, whereas phrases like *red balloon* are more transparent semantically, have phrasal stress, and pass the replacement by *one* test (as in, *I wanted a red balloon, but you gave me a blue one*). If an error contains part of a compound, the entire compound is included in the Example field (because this term must be at least the size of a word), and the error is treated as a morphological error affecting part of a word rather than an entire word.

English has many simple **clitics** that act like full words morpho-syntactically but are not full words phonologically. It is not difficult to distinguish clitics from affixes because both classes are relatively small sets, and their behavior is well-understood. In terms of their word status, clitics are treated as morpho-syntactic words, so for example, the erroneous insertion of the clitic *‘ve* in *I went* → *I’ve went* is treated as a word addition, rather than a morpheme error. Since they are phonologically dependent on a host, however, we include both the clitic and its host in the Example fields, because of the principle that these fields are at least the size of a word, where wordhood is determined both morphologically and phonologically.

Syntactic and morpho-syntactic errors also require reference to a host of information about English in the **Syntax variables**. These errors may violate one of several rules on morpho-syntactic features, such as the rule of subject-auxiliary verb agreement, which is assessed with the Form Rule Violation and Form Rule Type fields. Some errors are so-called transformation errors (Fay 1980), and the Transformation Type field classifies them by known English transformations, like the Particle Shift transformation. Several additional fields track and categorize other syntactic facts, such as agreement attraction, sentence complexity, and specific morpho-syntactic features. All of these are defined in detail in the OSF wiki.

Finally, there are a few facts that have not been made into fields in the database but are listed in the Notes field. As a result, the following **search terms** can be found in Notes to find records that exhibit these facts: attraction, over-regularization, over-tensing, affix stranding, accommodation, and allomorphy.

## Processing assumptions

Speech error classification can sometimes be a challenge because many speech errors exhibit **ambiguity** (Cutler 1988). In such cases, we employ a general strategy developed in Stemberger (1982/1985) of assembling all of the relevant evidence and using Occam’s Razor to pick the most likely analysis, and then record alternative types in the Alternative Mastertype field and explain our rationale in Notes. While speech errors may be ambiguous in many ways, the most common type involves sound and word ambiguity. For example, roughly 14% of phonological substitutions could be lexical substitutions (because the sound swap produces an actual word), and roughly 6% of lexical substitutions could be phonological substitutions (because the substituted word is phonologically similar). While favoring the most likely analysis may skew distributions of errors towards a dominant type, this ambiguity is represented in the database, and so it can be factored out in more careful experiments.

Several important assumptions shape the analysis of **the context of an error**. The conceptual framework for our coding of context is that speech errors arise through the perseveration or anticipation of words or sounds within a larger production plan because multiple linguistic units are activated simultaneously (Dell 1986; Levelt et al. 1999; Stemberger 1985). Thus, the activation of source words provides structure that can be occasionally mis-selected in a speech error. It is often the case that the context provides more than one potential source, and so to completely analyze the error, we again pick the best candidate using Occam’s Razor principles and at the same time mark all the potential sources in the longform entry. We distinguish source words from trigger words in deletion errors: trigger words contain structures that are identical to intended structure that is deleted (Shattuck-Hufnagel 1979). Finally, a fundamental question in describing the context of a speech error is how big the window is for finding source and trigger words. While some have argued for smaller windows (e.g., the six syllable window of Nooteboom (1969) for sound errors), our approach for all errors is to define windows that are ten words upstream and downstream of the error. The reason for this wider window is first, the theory behind speech error context is currently unclear, so a wider net opens up certain possibilities that are excluded with smaller windows. Additionally, our experience is that many examples have salient source words beyond Nooteboom’s smaller window. If future research determines that the contextual window for an error is smaller, then the window can easily be reduced programmatically given our consistent mark-up of the context.

Finally, **lexical substitutions** have been developed to certain standards that support a range of research. Lexical substitution errors tend to be semantically similar to the intended utterance (Dell et al. 1997; Jaeger 2005), and Word fields classify the errors into six semantic relationships, sub-types within these relationships, and the semantic field of intended-error pair. Following a technique used in Harley and MacAndrew (2001), lexical substitutions, as well as 200 control word pairs, were rated by 10 experimental participants on a 4-point scale of word relatedness, and mean ratings and reaction time are also given Word fields. Lexical substitutions often exhibit form similarity (Fay & Cutler 1977), and Word fields measure this similarity following known methods, including how form similarity is defined in the Philadelphia Naming Test (Roach et al. 1996) and some custom fields developed for this purpose. These Word fields are described in more detail in Alderete et al. (2023) and the OSF wiki.

## Conclusion

With this overview of the methods and logic of the database, SFUSED English can be used for a host of teaching and research activities. The speech error patterns can also be compared with those of other large English speech error collections (Fromkin 1971; Shattuck-Hufnagel 1979; Stemberger 1982/1985) to validate and extend these datasets. Future research can make use of the advantages of SFUSED English, including audio backup, time metrics, rich linguistic structure, and its large baselines, to ask new linguistic and psycholinguistic questions. Finally, the database is publicly available and can support a host of important but unexplored topics, including morphological errors, syntactic and morpho-syntactic errors, markedness effects on deletions and additions, production processing windows, and individual differences in error production.
